# Potential cultivation areas of *Euterpe edulis* (Martius) for rainforest recovery, repopulation and açai production in Santa Catarina, Brazil

**DOI:** 10.1038/s41598-023-32742-x

**Published:** 2023-04-18

**Authors:** Luiz Fernando de Novaes Vianna, Fábio Martinho Zambonim, Cristina Pandolfo

**Affiliations:** Centro de Informações de Recursos Ambientais e de Hidrometeorologia de Santa Catarina, Empresa de Pesquisa Agropecuária e Extensão Rural de Santa Catarina – Epagri, Rodovia Admar Gonzaga, 1347 – Itacorubi, Florianopolis, SC 88034-901 Brasil

**Keywords:** Agroecology, Agroecology

## Abstract

*Euterpe edulis* is an endangered palm species that provides the most important non-timber forest product exploited in its natural habitat, the Brazilian Atlantic Forest hotspot^1,4^. From 1991 to 2017, pasturelands, agriculture, and monoculture of tree plantations were responsible for 97% of Atlantic Forest deforested areas in Brazil and Santa Catarina was one of the Brazilian states with the greatest loss of forest area^14^. In the last decade, *E. edulis* fruits reached their highest commercial value, producing the southeastern equivalent of Amazonian ‘‘açai’’ (*Euterpe oleracea*)^5,7,8^. As a shade-tolerant species, *E. edulis* adapts very well to agroforestry systems^8,10^. To evaluate potential areas for cultivation of *E. edulis* through agroforestry systems, we developed and applied a spatial model for mapping suitable areas. To accomplish this, we analyzed multisource biophysical data and *E. edulis* distribution data from the Forest Inventory of Santa Catarina. We identified two areas with potential occurrence of the species, one in the domains of coastal Dense Ombrophilous Forest where the species is more common and another in the domains of inland Deciduous Seasonal Forest where its occurrence was suspected, but not proven, until 2021. Today, Deciduous Seasonal Forest is the most fragmented and impacted by agriculture. Our model, together with confirmed areas of occurrence, indicates that deciduous seasonal forest region should be prioritized for production and recovery of *E. edulis* through agroforestry systems.

## Introduction

Atlantic Forest is the natural habitat of *Euterpe edulis*, an endangered palm species^1^ biogeographically distributed across the Brazilian coast^2^, from sea level to 1000 m in altitude, both in steep slopes and dry forests^3^.

*E. edulis* is a slow-growing species, single-stemmed with no regrowth capacity, and its natural occurrence is dependent on well-preserved forests^4^. This palm species provides the most important non-timber forest product (NTFP) exploited in the Brazilian Atlantic Forest hotspot^5^. Palm heart extraction occurs by cutting adult individuals and subsequent removal of apical meristem, leading to the death of plants. Deforestation, the high degree of degradation of most forest remnants, and the pressure of palm heart exploration have all contributed to the risk of species extinction in the Atlantic Forest^4,6^.

In the last decade, *E. edulis* fruits reached their highest commercial value, producing the southeastern equivalent of Amazonian ‘‘açai’’ (*Euterpe oleracea*)^5,7,8^. The pulp of these palm fruits is rich in nutritional value, both anthocyanins and phenolic compounds, demonstrating high antioxidant capacity and thus currently used from a health and nutrition perspective^5,7–9^.

As a shade-tolerant species, *E. edulis* adapts very well to agroforestry systems (AST), an activity that has been growing and gaining in social and economic importance in the southern region of Brazil^8,10^.

AST is considered a sustainable land-use system that addresses production needs, while providing environmental benefits to society^11^. Agroforestry provides various ecosystem services, such as biodiversity conservation, carbon sequestration, reduction of crop diseases, increased biological controls, biological nitrogen fixation and nutrient cycling, besides income increments and food safety^8^. These systems have mechanisms to sustain high diversity of flora and fauna, thus driving conservation and improvement of diversity in agroecosystems^12^. AST maintains intermediate levels of biodiversity between natural forests and purely agricultural land-uses and could be used to increase connectivity in fragmented forest landscapes^13^. The high and complex levels of interaction between *E. edulis* and wild vertebrate animals suggest that this palm tree plays an important role in the successional dynamics of the forest ecosystem^12^.

From 1991 to 2017, pasturelands, agriculture, and monoculture of tree plantations were responsible for 97% of Atlantic Forest deforested areas in Brazil^14^. The consequences of Atlantic Forest habitat loss for biodiversity, ecosystem services, habitat destruction and loss of endemic species can be drastic, even for agriculture^14,15^. In Brazil, the dynamics of native rainforest cover, both loss and gain, are occurring in a heterogeneous manner across the six Brazilian biomes^14,16^. Despite the reduction in deforestation ratio from 1990 to 2015, the balance between loss and gain is still negative for forest cover maintenance and recovery, and Santa Catarina was one of the Brazilian states with the greatest loss of forest area in that period^16^. In Santa Catarina, forest loss is combined with growing forest fragmentation in some areas of Dense Ombrophilous Forest, Mixed Ombrophilous Fforest and Altitude Fields^14^.

Economically, strategies for forest recovery are necessary to reduce carbon emissions within the Brazilian Nationally Determined Contribution under the Paris Agreement—NDC^17^. One of the paths that can help reverse the deforestation scenario with less impact on agriculture is the gradual replacement of traditional agriculture with agroecological practices^18–20^.

The agroforestry production of “açai” from fruits of *E. edulis* represents a huge potential to reestablish connectivity in fragmented forest landscapes, generate income, contribute to food security for local family farmers and promote the conservation of the *E. edulis* by its economic use, while preserving it.

To evaluate potential areas for cultivation of *E. edulis* through AST, we developed and applied a spatial model for mapping areas where the ecological conditions are suitable for the natural occurrence of this palm tree. The objective was to guide agroecological public policies to stimulate and incentivize environmental recovery and food production with economical gains for the farmers.

To map suitable areas for *E. edulis*, we analyzed multisource biophysical data (physiography, climate, and vegetation cover) and *E. edulis* distribution data from FISC^21^ (https://www.iff.sc.gov.br/).

## Methods

Our spatial model was developed and implemented in R (https://www.r-project.org/) and ArcGis Desktop v. 10.6 (https://www.esri.com/en-us/arcgis/products/arcgis-desktop/overview) with the arcgisbiding (https://r.esri.com/assets/arcgisbinding-vignette.html), tidyverse (https://www.tidyverse.org/), corrplot (https://cran.r-project.org/web/packages/corrplot/vignettes/corrplot-intro.html), FactoMineR (https://cran.r-project.org/web/packages/FactoMineR/FactoMineR.pdf) and factoextra (https://cran.r-project.org/web/packages/factoextra/readme/README.html) packages. We divided it into three steps, including (1) the input and pre-processing of raw data (physiographic maps, climatic maps, Normalized Difference Vegetation Index (NDVI) and FISC *E. edulis* distribution data) in ArcGis; (2) the selection of variables to reduce multicollinearity; and (3) the application of the model using multicriteria and spatial analysis.

### Input and pre-processing data

**Figure 1:**
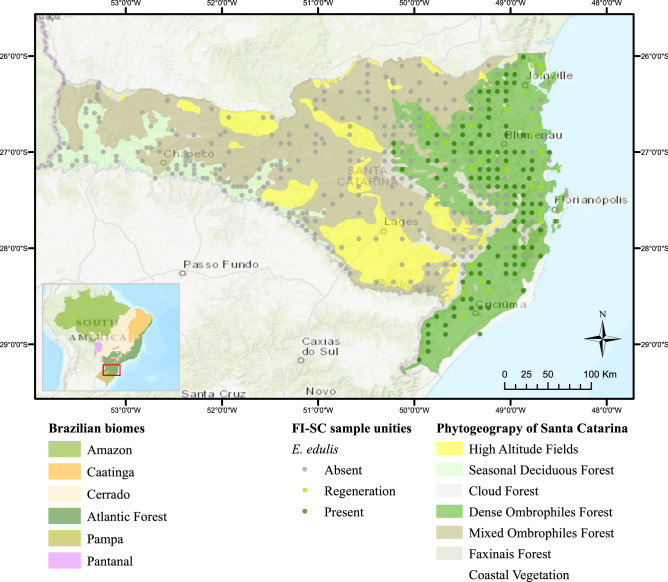
Brazilian biomes^31^, phytogeographical map of Santa Catarina^32^ and Santa Catarina Floristic Inventory sampling units^21^ with presence, absence, or regeneration of *E. edulis*.

For physiographic suitability analysis, we used the 90-m resolution Digital Elevation Model (DEM) of Shuttle Radar Topographic Mission (SRTM)^22^. From SRTM-DEM, we derived four terrain metrics: altitude (Alt), slope (Slp), topographic position index (TPI) and cosine of aspect (CosAspec)^23–25^. The climatic grids of annual mean of minimum temperatures (Tmin), annual mean temperatures (Tmea), annual mean of maximum temperatures (Tmax), annual number of frosts (NrFrost) and annual probability of frosts (ProbFrost) were also derived from SRTM-MDT, using regression equations from climatological data^26,27^. The potential annual insolation (Insol) grid was calculated using SRTM-DEM and the ArcGis Area Solar Radiation tool^28^. The grid of annual mean relative humidity (Humid) was obtained from the daily gridded meteorological variables in Brazil (1980–2013) database^29^. The annual mean and standard deviation of NDVI (NDVImean, NDVIstd) for the 2000–2019 period were calculated in ArcGis 10.6 using a Python 2.7 script^30^. The spatial distribution of 433 FISC^21^ sampling units (SU) with *E. edulis* presence, absence, or regeneration (Fig. 1) were used as sampling points for the model. We used the “extract multiple values to points” tool of ArcGis Spatial Analyst extension (https://desktop.arcgis.com/en/arcmap/10.6/tools/spatial-analyst-toolbox/extract-multi-values-to-points.htm) to populate the sampling points attribute table with all physiographic, climatic and NDVI data. Raw data and codes are available at https://doi.org/10.5281/zenodo.6620589. Results can be accessed at https://portal-arcgis.epagri.sc.gov.br/arcgis/home/index.html.

### Selection of variables to reduce multicollinearity

A multivariate analysis of variance (MANOVA) and a Bonferroni test were applied to evaluate the statistical difference of FISC^21^ sampling point groups (presence, absence, and regeneration of *E. edulis*) and to identify the variables responsible for this difference (Supplementary Table S2, Figure S1). A correlation analysis and a principal component analysis (PCA) with all physiographic, climatic and NDVI variables helped to select variables with the greatest influence on the differentiation of groups (Supplementary Figure S2) and reduce multicollinearity. Variables selected for the suitability model were those with lower correlation (≥ − 0.4 to ≤ 0.4) and higher loading (≥ 0.5) in the first four PC.

### Application of the model using spatial analysis

The suitability model was built by combining spatial analysis, multivariate statistics, multicriteria analysis, and map algebra^24,33,34^. A suitability map for each selected variable was generated by pixel reclassification. We defined the suitability criteria according to the mean and standard deviation of each variable by group (presence, absence, and regeneration). The raster dataset was reclassified for 1 (suitable) or 0 (unsuitable). Suitable pixels (1) were those with values within the mean and ± the standard deviation of the variable within the presence or regeneration SU groups. Pixel values deviating from this interval were classified as unsuitable (0) (Table 1).

**Table 1:** Suitability reclassification criteria for environmental variables and weight assigned to each map, according to the percentage of contribution in PC1.

Variable/Map	Acronym	Mean	Std	Suitable	Unsuitable	Weight
Altitude (m)	Alt	363.01	210.93	≤ 574	> 574	0.352
Annual mean temp. (°C)	Tmea	18.40	0.96	≥ 17.44	< 17.44	0.301
TPI	TPI	0.32	0.19	≤ 0.51	> 0.51	0.267
Annual insolation (h)	Insol	3838.39	273.26	≤ 4156.65	> 4156.65	0.031
Annual relative humidity (%)	Humid	81.50	0.57	≥ 80.93	< 80.93	0.028
Annual mean of NDVI	NDVImean	83.76	3.98	≥ 79.72	< 79.72	0.012

A weighted sum of all suitability maps resulted in the final suitability map.

Suitability map = (Alt × 0.352) + (Tmea × 0.301) + (TPI × 0.267) + (Insol × 0.031) + (Humid × 0.028) + (NDVImean × 0.012).

Suitability maps were integrated by map algebra in ArcGis. We executed a second PCA with the selected variables to evaluate the influence of each variable (PCA contribution) on grouping. The contribution value of each variable on the first PC was used to weight maps (Supplementary Figure S3-A) (Table 1).

## Results

The three SU groups are statistically different according to environmental variables evaluated (Supplementary Table S1, Figure S1). The group with presence of *E. edulis* was differentiated from the absent group by all variables, except terrain orientation (cosine of aspect) (Supplementary Figure S1). The SU of the regeneration group can have environmental characteristics similar to those of both presence and absence groups, but it is completely within the presence group (Supplementary Figure S1).

*E. edulis* presence and regeneration groups occur in the SU with lower altitudes (mean 363 m and 271 m) compared to altitudes of the SU where the species does not occur (mean 776 m) (Supplementary Table S2). The mean temperatures of the SU with *E. edulis* are higher (18.4 °C and 19.2 °C) than those observed in the SU without the species (17 °C). This is also observed for the number of frosts, which is rarer in the SU where *E. edulis* was found (Supplementary Table S2). Terrain position (TPI) also seems to correlate with the presence of *E. edulis*, which was observed more frequently in valleys and lower slopes (TPI < 0.32), but rarer on upper slopes and hilltops (TPI > 0.51). Other variables were also correlated with the presence of *E. edulis*, such as relative humidity and NDVI, which also showed higher mean values than the values observed in the SU without the species (Supplementary Table S2).

In the second PCA (Supplementary Figure S3), the variables altitude, TPI and average temperature obtained the greatest loadings in the first PC, which explained 43.7% of data variability. The variables relative humidity, NDVI mean, and annual insolation were those with the greatest loadings in the second PC, which explained 21.9% of data variability. The third PC (15.2% of data variability) also had the variables insolation and relative humidity as those with the greatest loadings.

**Figure 2:**
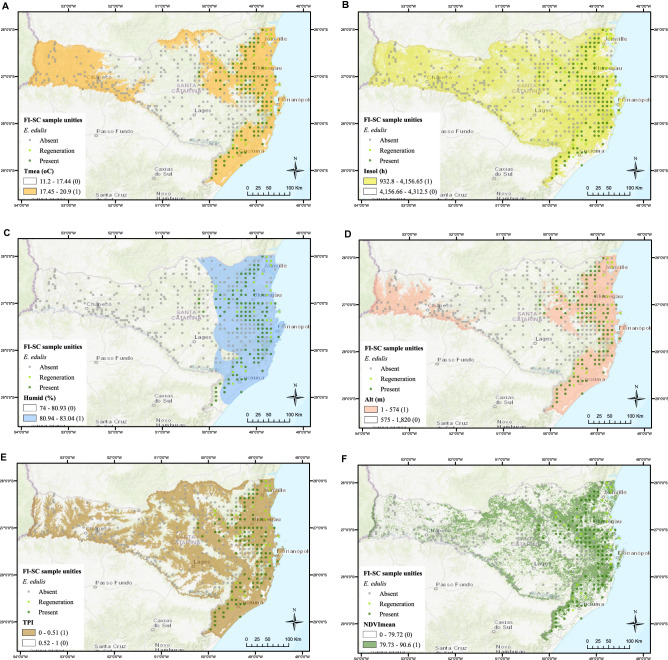
Spatial distribution of the Floristic Forest Inventory of Santa Catarina sampling units with *E. edulis* by (**A**) annual mean temperature; (**B**) annual potential insolation; (**C**) annual mean relative humidity; (**D**) altitude; (**E**) topographic position index; and (**F**) annual mean of NDVI.

Climatically, the SU with *E. edulis* predominates in areas with mean annual temperatures higher than 17.44 °C (Fig. 2A), annual insolation lower than 4156.65 h (Fig. 2B), and relative humidity higher than 80.93% (Fig. 2C). Suitable physiographic sites for *E. edulis* are those with altitude lower than 574 m (Fig. 2D) and topographic positions of lower slopes and valleys (TPI ≤ 0.51) (Fig. 2E). According to vegetation index, E. edulis is associated with the presence of forest remnants (annual mean of NDVI greater than 79.72) less fragmented (Fig. 2F). These variables were indicated for mapping the suitable homogeneous regions for the natural occurrence of *E. edulis* (Table 1, Fig. 2).

**Figure 3:**
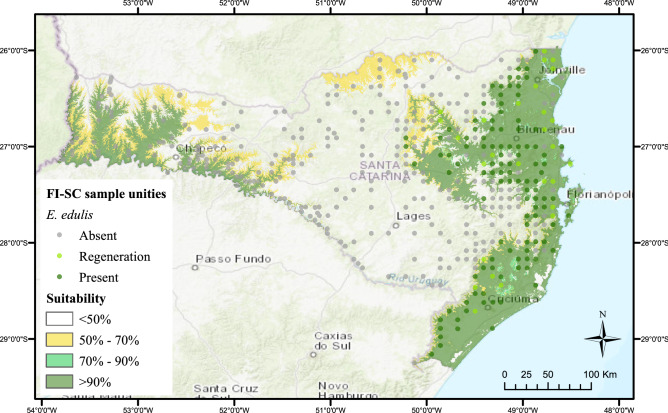
Spatial distribution of the Floristic Forest Inventory of Santa Catarina sampling units with *E. edulis* by suitability.

Two continuous suitable areas (Fig. 3—suitability > 90%) for natural presence of *E. edulis* were identified by the model. These areas are within the domains of dense ombrophilous forest (eastern green area) and seasonal deciduous forest (western green area) (Figs. 1 and 3). Together, the suitable areas cover 29,717km2, or 31% of Santa Catarina territory. All SUs with *E. edulis* are within dense ombrophilous forest in the eastern area (Fig. 3).

## Discussion

The first scientific publications on the geographic distribution of *E. edulis* in Santa Catarina included studies conducted between 1969 and 1972^35,36^. The authors cited the surveys of *E. edulis* carried out between 1949 and 1953. The only region confirmed with the presence of the species, until then, was the dense ombrophilous forest domain (Figs. 1 and 3—eastern green area). Despite reference to the rare occurrence of *E. edulis* in the Uruguay River valley (Fig. 3—western green area), a region of seasonal deciduous forest (Fig. 1), the authors state that no specimens were found in field surveys.

The authors^35,36^ believed that it was possible to find *E. edulis* also in the deciduous seasonal forest domains, as was indicated by our model (Fig. 3). However, this confirmation only occurred recently^37^. The newest records of *E. edulis* in natural forest fragments in western Santa Catarina help to confirm the suspicion of the former botanists, as well as validate the model of potential areas. The fragments of seasonal deciduous forest are smaller, sparser, and almost discontinuous when compared to those of dense ombrophilous forest (Fig. 3F). Habitat loss at deciduous seasonal forest explains the scarcity of individuals of *E. edulis*, even though it is a region ecologically suitable for the species^6,37^.

The ecological suitability presented by the model, the scarce presence of *E. edulis* individuals, and number of forest fragments all make the deciduous seasonal forest region a priority for investment in agroforestry systems that encourage the recovery of native forest and açai production.

Historically, agriculture in the western region of Santa Catarina changed from an agroecological subsistence system practiced by traditional communities (indigenous and caboclos) to a capitalist model implemented by European immigrants from Germany, Italy, and Poland^38^. After colonization, wood exploitation was one of the main economic activities of the state. Between the decades of the 1940s and 1960s, the wood obtained from native forests represented about half of Santa Catarina’s total exports, resulting in an alarming decrease in original forest cover^36^, especially the domains of deciduous seasonal forest^39^.

From the 1960s to now, the model based on increasing grain productivity (soybean and corn) and the expansion of pig, poultry and dairy farming contributed to deforestation, soil degradation^37,38^ and, mainly, the reduction in water sources and contamination^39^. Currently, both floristic composition and fauna hold predominance as secondary species, a fact that illustrates the historic exploitation and degradation of deciduous seasonal forest in the state^40^.

Striking a balance between personal economic gain for the rural producer and stewardship of the ecosystem that favors the production of quality food, water supply, soil maintenance, and biodiversity increase is a challenge for agroforestry policies^41,42^. To provide a transition to a new agroecological approach in the western region of Santa Catarina, all actors must cooperate in forging a long-term project^43^, and sound public policy is crucial to accomplish this^42,44^.

To help stakeholders in choosing critical or priority areas to increase agroecosystem biodiversity, connect forests fragments, and minimize the risk of *E. edulis* extinction, while, at the same time, producing fruits through agroforestry, all data and maps produced in this study are available in https://portal-arcgis.epagri.sc.gov.br/arcgis/home/index.html.

## Supplementary Information

Supplementary Information.

## Data Availability

All our dataset and codes are available from Zenodo (https://doi.org/10.5281/zenodo.6620589) under the Creative Commons Attribution 4.0 International Public License.
