# Supplementary material for: Potential cultivation areas of Euterpe edulis (Martius) for rainforest recovery, repopulation and açai production in Santa Catarina, Brazil
Source: Sci Rep. 2023 Apr 18;13:6272. doi: 10.1038/s41598-023-32742-x (PMC10113375; doi:10.1038/s41598-023-32742-x)
Supplement: Supplementary file 1 — Supplementary Information. [file 41598_2023_32742_MOESM1_ESM.docx]

**Supplementary information for “Potential cultivation areas of *Euterpe edulis* (Martius) for rainforest recovery, repopulation and açai production in Santa Catarina, Brazil”**

Table S1: MANOVA result.

| Df | Pillai | approx. F | num Df | den Df | Pr(>F) |
| --- | --- | --- | --- | --- | --- |
| CompEuterpe | 2 | 0.73206 | 18.564 | 26 | 836 < 2.2e-16 *** |
| Residuals | 429 |  |  |  |  |

Signif. codes: 0 ‘***’ 0.001 ‘**’ 0.01 ‘*’ 0.05 ‘.’ 0.1 ‘ ’ 1

Variables and Acronyms

Altitude (Alt)

Slope (Slope)

Topographic position index (TPI)

Cosine of aspect (CosAspec)

Annual mean of minimum temperatures (Tmin)

Annual mean temperatures (Tmea)

Annual mean of maximum temperatures (Tmax)

Annual number of frosts (NrFrost)

Annual probability of frosts (ProbFrost)

Potential annual insolation (Insol)

Annual mean relative humidity (Humid)

Annual mean of NDVI (NDVImean)

Annual standard deviation of NDVI (NDVIstd)

Figure S1. Bonferroni test result. Each graph represents one variable. The red pairwise groups are different (p<0.5) considering the corresponding variable average. Present and absent groups are distinguishable by almost all variables, except cosine of aspect. Regeneration and absent groups are not distinguishable by slope, insolation and NDVI variables. Regeneration and present groups are distinguishable only by six variables.


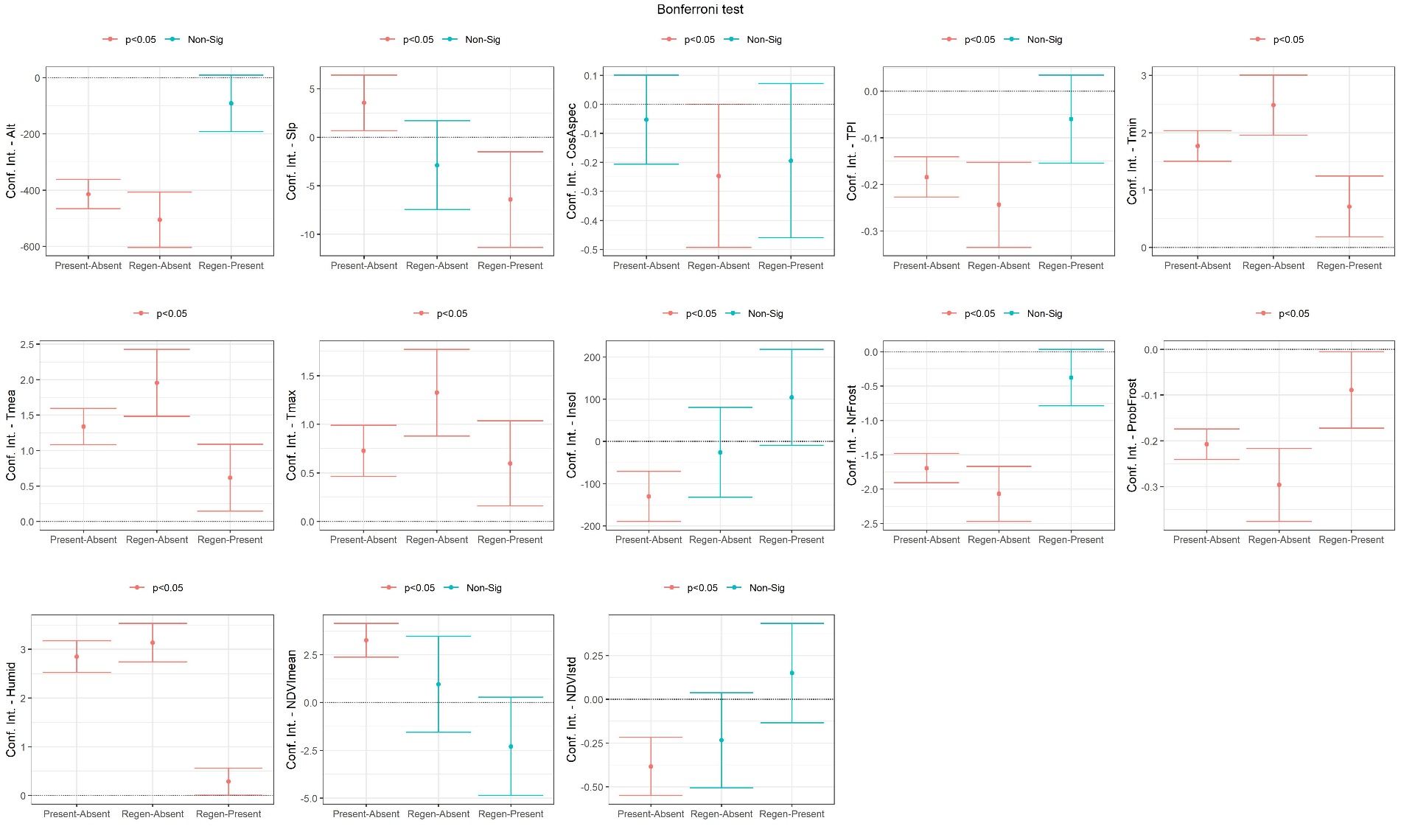


| Table S2: Descriptive statistics of variables in sampling units by *E. edulis* presence status (groups). | | | | | | | |
| --- | --- | --- | --- | --- | --- | --- | --- |
| **Sample Units with absence of *E. edulis*** | | | | | | | |
|  | n | Mean | Std. Dev. | Median | Min. | Max. | Range |
| Altitude (m) | 297 | 776.86 | 292.27 | 806.00 | 8.00 | 1583.00 | 1575.00 |
| Slope (%) | 297 | 20.94 | 13.09 | 19.33 | 0.81 | 66.40 | 65.59 |
| Cosine of Aspec | 297 | -0.01 | 0.71 | -0.01 | -1.00 | 1.00 | 2.00 |
| TPI | 297 | 0.51 | 0.22 | 0.52 | 0.01 | 0.95 | 0.93 |
| Annual mean of minimum temperature (^o^C) | 297 | 12.83 | 1.56 | 12.87 | 8.12 | 17.37 | 9.25 |
| Annual mean temperature (^o^C) | 297 | 17.06 | 1.59 | 16.98 | 12.32 | 20.86 | 8.54 |
| Annual mean of maximum temperature (^o^C) | 297 | 23.13 | 1.73 | 22.94 | 18.16 | 27.02 | 8.85 |
| Annual insolation (h) | 297 | 3968.65 | 254.72 | 4006.02 | 2957.45 | 4312.50 | 1355.05 |
| Annual mean number of frost (n) | 297 | 3.65 | 1.20 | 3.77 | 0.50 | 6.95 | 6.45 |
| Annual probability of frost | 297 | 0.82 | 0.14 | 0.86 | 0.25 | 0.97 | 0.72 |
| Annual relative humidity (%) | 296 | 78.65 | 2.68 | 79.40 | 74.04 | 82.92 | 8.87 |
| Annual mean of NDVI | 297 | 80.50 | 4.10 | 81.50 | 66.06 | 88.64 | 22.58 |
| Annual standard deviation of NDVI | 297 | 2.59 | 0.89 | 2.44 | 0.94 | 6.60 | 5.67 |
| **Sample Units with presence of *E. edulis*** | | | | | | | |
|  | n | Mean | Std. Dev. | Median | Min. | Max. | Range |
| Altitude (m) | 109 | 363.01 | 210.93 | 322.00 | 7.00 | 923.00 | 916.00 |
| Slope (%) | 109 | 24.48 | 12.88 | 24.42 | 2.00 | 77.01 | 75.00 |
| Cosine of Aspec | 109 | -0.07 | 0.69 | -0.20 | -1.00 | 0.99 | 1.99 |
| TPI | 109 | 0.32 | 0.19 | 0.29 | 0.02 | 0.87 | 0.85 |
| Annual mean of minimum temperature (^o^C) | 109 | 14.60 | 1.05 | 14.68 | 12.58 | 16.93 | 4.35 |
| Annual mean temperature (^o^C) | 109 | 18.40 | 0.96 | 18.48 | 16.44 | 20.49 | 4.05 |
| Annual mean of maximum temperature (^o^C) | 109 | 23.85 | 0.91 | 23.81 | 22.15 | 25.96 | 3.81 |
| Annual insolation (h) | 109 | 3838.39 | 273.26 | 3892.59 | 2785.19 | 4280.82 | 1495.63 |
| Annual mean number of frost (n) | 109 | 1.95 | 0.86 | 1.78 | 0.49 | 4.25 | 3.75 |
| Annual probability of frost | 109 | 0.61 | 0.16 | 0.61 | 0.25 | 0.89 | 0.64 |
| Annual relative humidity (%) | 109 | 81.50 | 0.57 | 81.40 | 80.04 | 82.65 | 2.61 |
| Annual mean of NDVI | 109 | 83.76 | 3.98 | 84.69 | 71.20 | 89.32 | 18.12 |
| Annual standard deviation of NDVI | 109 | 2.21 | 0.70 | 2.19 | 0.80 | 5.30 | 4.50 |
| **Sample Units with regeneration of *E. edulis*** | | | | | | | |
|  | n | Mean | Std. Dev. | Median | Min. | Max. | Range |
| Altitude (m) | 27 | 271.63 | 234.63 | 185.00 | 10.00 | 780.00 | 770.00 |
| Slope (%) | 27 | 18.07 | 11.00 | 18.11 | 1.39 | 35.95 | 34.56 |
| Cosine of Aspec | 27 | -0.26 | 0.59 | -0.41 | -1.00 | 0.99 | 1.99 |
| TPI | 27 | 0.26 | 0.22 | 0.18 | 0.01 | 0.77 | 0.76 |
| Annual mean of minimum temperature (^o^C) | 27 | 15.31 | 1.25 | 15.26 | 12.79 | 17.32 | 4.53 |
| Annual mean temperature (^o^C) | 27 | 19.02 | 1.11 | 19.14 | 16.69 | 20.79 | 4.10 |
| Annual mean of maximum temperature (^o^C) | 27 | 24.45 | 1.02 | 24.35 | 22.37 | 26.19 | 3.82 |
| Annual insolation (h) | 27 | 3942.93 | 259.57 | 4037.63 | 3176.59 | 4238.17 | 1061.58 |
| Annual mean number of frost (n) | 27 | 1.58 | 0.96 | 1.22 | 0.51 | 3.66 | 3.15 |
| Annual probability of frost | 27 | 0.52 | 0.20 | 0.48 | 0.25 | 0.85 | 0.60 |
| Annual relative humidity (%) | 27 | 81.79 | 0.65 | 81.64 | 80.94 | 82.87 | 1.94 |
| Annual mean of NDVI | 27 | 81.46 | 6.25 | 84.64 | 63.38 | 87.71 | 24.34 |
| Annual standard deviation of NDVI | 27 | 2.36 | 0.64 | 2.44 | 0.83 | 3.80 | 2.97 |

Figure S2. Correlation matrix result (CorrPlot) and first PCA result: all variables

| A. Corrplot | B. PCA Scree Plot (Autovalues) |
| --- | --- |
| 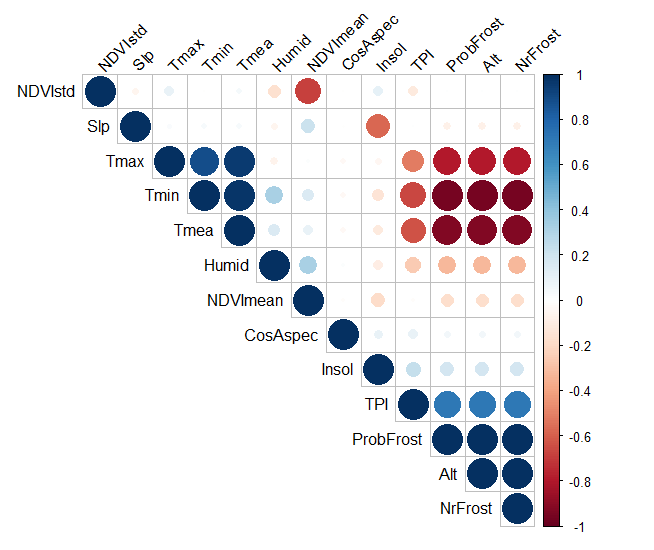 | 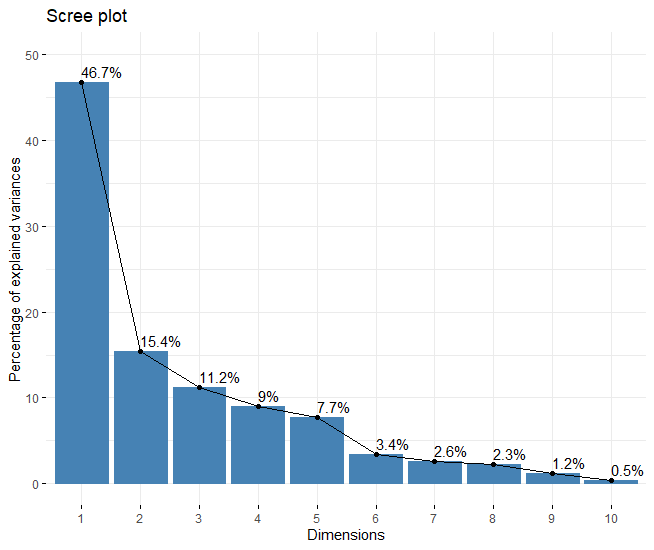 |
| C. PCA Loadings | D. PCA Biplot |
| 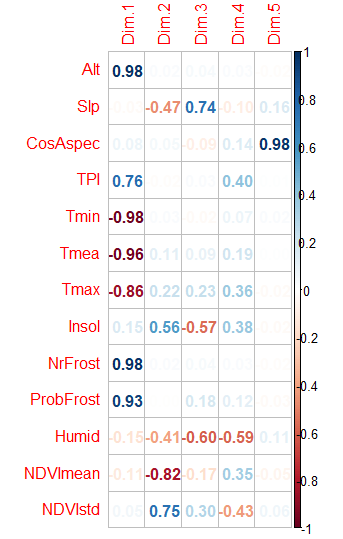 | 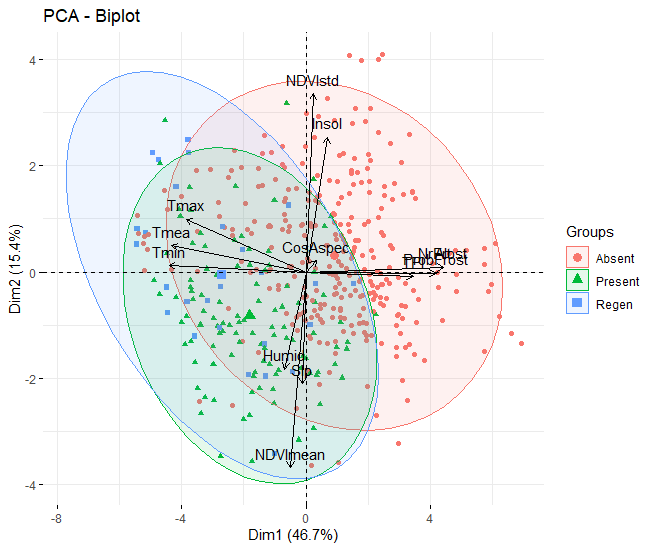 |

Figure S3. Second PCA result: selected variables

| A. PCA contribution | B. PCA Scree Plot (Autovalues) |
| --- | --- |
| \| Variable \| Dim.1 \| Dim.2 \| Dim.3 \| Dim.4 \| Dim.5 \| \| --- \| --- \| --- \| --- \| --- \| --- \| \| Alt \| 35.21 \| 1.16 \| 0.15 \| 2.04 \| 7.07 \| \| Tmea \| 30.16 \| 6.58 \| 1.22 \| 7.02 \| 12.48 \| \| TPI \| 27.67 \| 0.40 \| 0.96 \| 10.08 \| 59.94 \| \| Insol \| 3.12 \| 21.64 \| 44.79 \| 26.07 \| 4.35 \| \| Humid \| 2.77 \| 28.71 \| 50.97 \| 7.19 \| 8.28 \| \| NDVImean \| 1.07 \| 41.50 \| 1.91 \| 47.60 \| 7.89 \| | 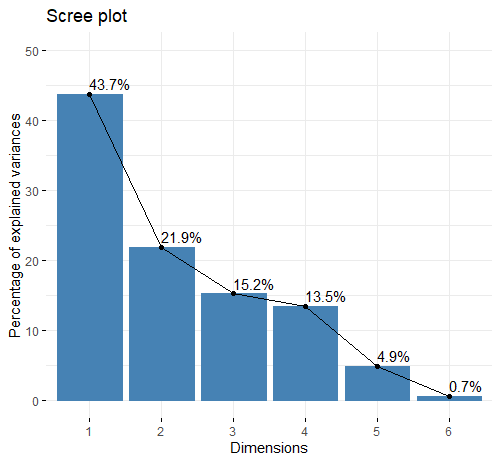 |
| C. PCA Loadings | D. PCA Biplot |
| 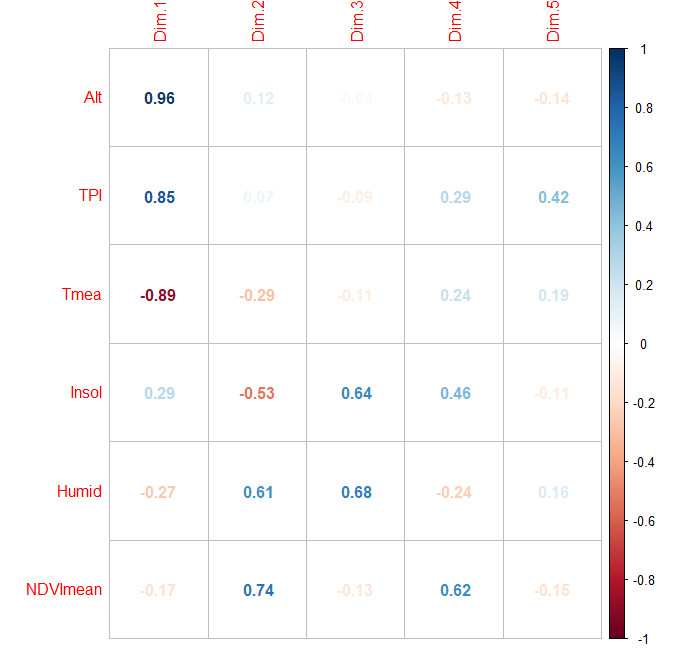 | 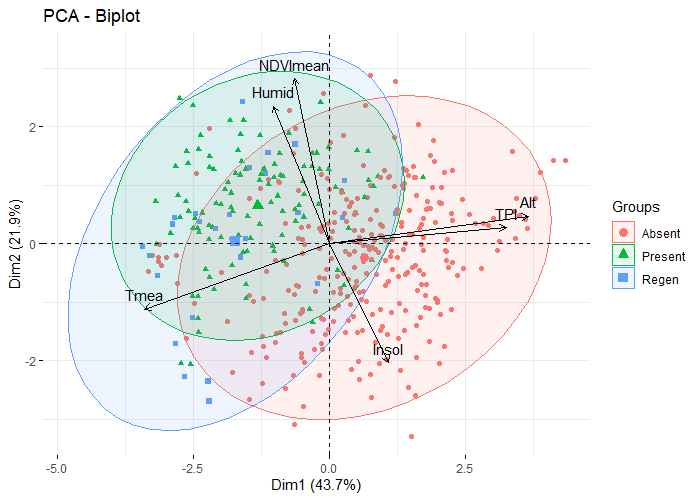 |
